# The Influence of Inulin on the Retention of Polyphenolic Compounds during the Drying of Blackcurrant Juice

**DOI:** 10.3390/molecules24224167

**Published:** 2019-11-17

**Authors:** Anna Michalska, Aneta Wojdyło, Jessica Brzezowska, Joanna Majerska, Ewa Ciska

**Affiliations:** 1 Department of Fruit, Vegetable and Plant Nutraceutical Technology, the Faculty of Biotechnology and Food Science, Wrocław University of Environmental and Life Sciences, ul. Chełmońskiego 37, 51-630 Wrocław, Poland; aneta.wojdylo@upwr.edu.pl (A.W.); jessica.brzezowska@upwr.edu.pl (J.B.); 2 Department of Chemistry and Biodynamics of Food, Institute of Animal Reproduction and Food Research of the Polish Academy of Sciences in Olsztyn, ul. Tuwima 10, 10-748 Olsztyn, Poland; e.ciska@pan.olsztyn.pl; 3 Institute of Agricultural Engineering, the Faculty of Life Sciences and Technology, Wrocław University of Environmental and Life Sciences, ul. Chełmońskiego 37a, 51-630 Wrocław, Poland; joanna.majerska@upwr.edu.pl

**Keywords:** *Ribes nigrus* L., juice, powders, carriers, polyphenols, hydroxymethyl-l-furfural

## Abstract

In blackcurrant juice powders made using freeze-, vacuum-, and spray-drying methods, 19 polyphenolic compounds were identified: anthocyanins (6), (+)-catechin, flavonols (8), and phenolic acids (4). The highest content of identified polyphenols was noted after vacuum drying at 90 °C, which was connected with the thermally induced release of (+)-catechin. Drying at this temperature also increased the formation of the Maillard reaction/caramelization product, hydroxymethyl-l-furfural, when inulin was added. The higher the vacuum drying temperature was, the stronger the degradation of anthocyanins was. Inulin was a better protectant of anthocyanins than maltodextrin, except during vacuum drying at 90 °C, which probably triggered inulin’s participation in the formation of hydroxymethyl-l-furfural (HMF), thus limiting its capability to protect anthocyanins. Flavonols and phenolic acids were best retained after vacuum drying at 50 °C. Carrier selection affected only slightly, whereas carrier concentration did not affect, the content of flavonols and phenolic acids. The quality of fruit juice powders should be considered taking into account a broad spectrum of factors, including the initial composition of the material subjected to drying, the drying parameters, the carrier type and concentration, and the interactions that occur during the thermal treatment of fruit juices.

## 1. Introduction

Blackcurrant (*Ribes nigrum* L.) is a fruit shrub from the Grossulariaceae family originating from the European and Asian areas situated in the temperate climate zone [1]. In 2016, the global blackcurrant production was 655.03 thousand tons, with the top producers being Russia (60.3%), Poland (25.4%), Ukraine (3.7%) and Germany (2.1%) [2]. As almost the entire blackcurrant production in Russia covers the needs of the internal market, it is Poland which is considered internationally as the largest blackcurrant exporter. Unfortunately, there has recently been an overproduction of blackcurrant, so its production is becoming less and less profitable, and the fruit is not fully utilized. But blackcurrant is a very valuable material thanks to its high health-promoting potential, which results from the presence of bioactive compounds, including high amounts of vitamin C and polyphenols [3]. Besides many healthy chemical compounds, blackcurrant also contains a lot of dietary fiber, such as pectin [4], and has a high profile of organic acids [5] and mineral compounds such as potassium, phosphorus, calcium, and magnesium [6]. All these properties not only contribute to the sensory advantages of blackcurrant, but they also determine a number of health-promoting features, including anticarcinogenic, antibacterial, and anti-inflammatory effects or neuro- or cardioprotective activities [7,8,9]. In view of the blackcurrant oversupply relative to its demand on the market on the one hand, and the blackcurrant’s chemical composition resulting in its excellent health-supporting potential on the other, new solutions are being sought mainly in the food, pharmaceutical, and cosmetic industries that would enable using this fruit alternatively to fresh consumption. Among the novel products available for sale, one can already find candied blackcurrants [10], functional concentrate-based candies [11], blackcurrant-enriched beer [12], freeze-dried and powdered products [13,14], or blackcurrant-based pigments used in hair dyes [15].

Another innovative method to utilize the potential of this fruit is the production of fruit powders, which—if obtained using proper drying methods and parameters—may be a highly attractive product in terms of quality and versatility. Fruit powders are an excellent colorant, a great taste booster, and they additionally enrich final food products with components beneficial to the human body [16]. This makes them a valuable ingredient in drinks, yogurts, ice creams or sugar confectionary products, as well as an additive in, sauces, extruded cereal products, or foods for children [17]. Fruit powders can be made from the whole fruit, fruit waste (pomace), juices, or concentrates. The last two types of products pose a challenge to food science when it comes to applying a proper drying technique, mainly because of their chemical composition [16]. The low glass transition temperature, high hygroscopicity, low melting temperature, and high solubility in water of the main juice components (low-molecular sugars and organic acids) render fruit juices viscous and, thus, very difficult to dry [17,18]. The most popular drying methods used to obtain fruit juice powders are freeze-drying, regarded as having the mildest effect on thermolabile compounds, vacuum drying, and spray drying [19]. The typical carriers that enable obtaining fruit powders are: gum arabic, maltodextrins with different dextrose equivalents, gelatin, starch, pectin, methylcellulose, alginates, and tricalcium phosphate and their combinations. Their addition significantly shapes the final physical and chemical properties of powders [16,20]. A potential innovative carrier is inulin, a polysaccharide from the group of nondigestive carbohydrates called fructans. In 2003, inulin received a generally recognized as safe (GRAS) status [21] and has been found to be present in about 36,000 plant species, among which chicory is considered to be the richest inulin source. Because of its high solubility in hot water, neutral smell, good gelling, water absorption, thickening and emulsifying capabilities, and its positive effect on the digestive system, inulin is commonly applied as a functional food additive, including as a prebiotic, a fat or sugar substitute, or a texture modifier [21].

An incorrect selection of process parameters may lead to a significant degradation of valuable nutritious and bioactive compounds and, thus, to a low quality of the final product. Therefore, it is important to select a drying method and a carrier that will, as best as possible, prevent harmful processes during fruit powder production and, thus, enable obtaining a product with optimal physical and chemical properties [18,22]. The literature provides little information about interactions between juice components and carriers triggered by different drying methods. Therefore, the aim of the study was to analyze the influence of inulin and its mixtures (maltodextrin:inulin) on the qualitative and quantitative changes in the composition of polyphenolic compounds in blackcurrant juice submitted to freeze-, vacuum- (at 50, 70, and 90 °C), and spray-drying methods. The study also included an assessment of the influence of the carrier and the drying parameters on the formation of hydroxymethylfurfural and alterations in the antioxidant capacity of the obtained products.

## 2. Results and Discussion

Processing of blackcurrant into juice leads to alterations in the content of bioactive components, especially polyphenolic compounds [23,24]. Further handling of juice, including drying, can modify the composition and the quantity of polyphenols as a result of different factors, such as the initial composition of the juice, the applied drying processes and parameters, the added carrier and its concentration, and, most importantly, the interactions between the bioactive compounds themselves and between the bioactives and the carriers [16,25,26,27]. In the current study, carriers were chosen based on popularity (maltodextrin) and functionality (inulin), and their mixtures were supposed to improve the drying properties of inulin [28]. The concentration of the carriers was chosen based on a preliminary experiment designed to select carrier concentrations that can be used to produce fine powders by all drying methods applied in study.

### 2.1. Polyphenolic Compounds

Four major groups of polyphenolic compounds were identified in blackcurrant juice powders: anthocyanins, flavan-3-ols, flavonols, and phenolic acids. All of them were previously reported to be present in blackcurrant products [23,24,29]. The sum of polyphenolic compounds identified in the analyzed powders ranged from 1.8 g/kg, of dry basis (db) in the freeze-dried product containing 40% of the maltodextrin:inulin (2:1) mixture, to 56.1 g/kg db, in the product obtained after vacuum drying at 90 °C containing 30% of inulin ( Table 1, Table 2 and Table 3). The average content of polyphenolic compounds in freeze-dried powders ($\bar{x}$ = 2.3 g/kg db) was approximately 12.6 times lower when compared to that observed after vacuum drying at 90 °C, regardless of the carrier type and concentration. This was due to a significant release of (+)-catechin during vacuum drying at 90 °C (as described below). Contrary to powders made from blackcurrant extract using spray drying [27], spray dried juice powders with the addition of inulin had the highest sum of identified polyphenolic compounds: their levels were, respectively, 21%, 43%, and 48% higher than that in powders containing the maltodextrin:inulin (2:1) and maltodextrin:inulin (3:1) mixtures as well as maltodextrin alone. Thus, the study confirmed that appropriate carrier selection could significantly improve the retention of polyphenolic compounds in blackcurrant powders.

A more detailed analysis revealed that of all identified groups of polyphenolic compounds present in blackcurrant juice powders, anthocyanins were the most abundant [27,29] (46.3% of all polyphenolic compounds). Their content ranged from 75.9 mg/kg db, in powders obtained after vacuum drying at 90 °C and containing 30% of inulin, to 3375.2 mg/kg db, in products obtained after vacuum drying at 70 °C and containing a mixture of maltodextrin and inulin (2:1; *w*/*w*) (Table 1).

Similarly to previous studies [29,30,31], the major anthocyanins were cyanidin- and delphinidin-3-*O*-rutinosides. Their percentage shares in the total content of identified anthocyanins in the analyzed powders were 46.1% and 36.4%, respectively, followed by delphinidin-3-*O*-glucoside (14.2%), petunidin-3-*O*-rutinoside (1.7%), peonidin-3-*O*-rutinoside (1.3%), and cyanidin-3-*O*-glucoside (0.2%). The drying processes had a stronger impact on anthocyanin content than the carrier type and concentration. The average content of anthocyanins in powders, regardless of the type and concentration of the added carrier, was the highest after vacuum drying at 50 °C (2354.7 mg/kg db) and 70 °C. Previously, it was proved that applying a temperature of 50 °C could lead to the deactivation of polyphenol oxidase and, thus, could contribute to a better retention of these compounds [32]. This finding was similar to the observation made for blackcurrant pomace dried by convection at 50 °C [14]. In this study, the lowest content of anthocyanins was noted after vacuum drying at 90 °C—it was almost 2.5 times lower when compared to that observed after vacuum drying at 50 °C. So the results of this study were in agreement with the previous studies on blackcurrant, suggesting a strong influence of temperature on the degradation of anthocyanins during drying [32,33].

As far as the type of carrier is concerned, the highest retention of anthocyanins was noted when the mixture of maltodextrin and inulin (2:1 and 3:1, *w*/*w*) was applied. As for adding a single carrier, a stronger degradation of anthocyanins was observed when maltodextrin was used—it was, on average, almost 27.6% lower when compared to that identified after adding inulin for all drying methods except vacuum drying at 90 °C. This was in contrast to Lima et al. [34] and Bakowska-Barczak and Kołodziejczyk [27]. The different results obtained in the latter study could result from the fact that the moisture content of powders made using different carriers was not considered, and the results were expressed per fresh weight. On the other hand, the degradation of anthocyanins in powders containing inulin was stronger than in powders containing maltodextrin after vacuum drying at a high temperature (i.e., 90 °C). A possible explanation is that, at this temperature, inulin takes part in the formation of Maillard reaction/caramelization products [35], including hydroxymethyl-l-furfural, in that hydroxymethyl-l-furfural (HMF) is formed of fructose present in inulin. As fructose moieties participate in the formation of HMF [36], its content might significantly increase after vacuum drying at 90 °C (Table 4), and consequently inulin might no longer protect anthocyanins from the degradation initiated by high temperature. The concentrations of the carriers (30%, 35%, and 40%) influenced the content of anthocyanins, but to a lower extent when compared to the drying method and carrier type used.

The second group of polyphenolic compounds identified in blackcurrant juice powders was flavan-3-ols, represented by (+)-catechin (43.2% of all polyphenols), the percentage share of which ranged from 0% (freeze drying) to 96.5% (vacuum drying at 90 °C) (Table 1). Among the rest of the applied drying methods, vacuum drying at 50 °C resulted in the lowest content of (+)-catechin, which was almost 130 times lower when compared to vacuum drying at 90 °C. This strongly suggests the heat-induced release of (+)-catechin from polymerized structures during the drying of blackcurrant juice. A similar observation was made in the case of roasting cocoa beans [37]. Such controlled (temperature-dependent) release of selected polyphenolic compounds might help to design the final content of bioactives in powdered products. As far as the type of carrier is concerned, maltodextrin seems to limit the release of (+)-catechin during vacuum drying at 50 and 70 °C and during spray drying, whereas the content of this flavan-3-ol in powders vacuum dried at 90 °C with the addition of inulin was almost 4 times higher when compared to powders vacuum dried at 90 °C with the addition of maltodextrin. As previously mentioned, inulin may take part in HMF formation, so its share in the blackcurrant juice subjected to drying may decrease, thus improving the release of (+)-catechin.

Flavonols were the third group of polyphenolic compounds present in the analyzed powders (8% of all polyphenolic compounds), 8 compounds of which were identified with the following contribution: quercetin-3-*O*-rutinoside > myricetin-3-*O*-galactoside > derivative of flavonol (**1**) > quercetin-3-*O*-malonylglucoside > myricetin-3-*O*-rutinoside > quercetin-3-*O*-glucoside > kaempferol-3-*O*-rutinoside > derivative of flavonol (**2**) (Table 2) [38]. Individual flavonols present in blackcurrant juice powders were similar to those present in powders made from blackcurrant pomace [13], but their content in the juice powders was influenced by the juicing process [3].

The drying methods applied in this study caused alterations in the sum of flavonols, which was the highest after vacuum drying at 50 and 70 °C and the lowest after vacuum drying at 90 °C. This is in agreement with the observation made during the drying of blackcurrant pomace, where an increase in temperature above 80 °C resulted in a significant decrease in the content of flavonols. The carriers had a smaller impact on the content of flavonols than the drying methods. Contrary to juice powders made from plum [16] and cranberry [39], the concentration of carriers added to juice before drying had no statistically significant influence on the sum of flavonoids in the products.

The group with the smallest share in the sum of polyphenolic compounds consisted of 4 phenolic acids (2.5% of all polyphenolic compounds). Chlorogenic acid and *p*-coumaric acids were the dominant compounds in the group, and their contributions in the powders were 60% and 25.3%, respectively (Table 3). Previously, gallic and *p*-coumaric acids were found to be dominant in blackcurrant juice [3], whereas Pinelo et al. [40] identified neochlorogenic acid, but did not confirm the presence of gallic acids. Thus, the content and presence of individual phenolic acids might differ depending on the cultivar and juice preparation [3,23,31]. Neochlorogenic acid and derivatives of *p*-coumaric acids were also identified in the current study (Table 3), but they were not found in blackcurrant pomace powders [13].

As in the case of flavonols, vacuum drying at 70 and 90 °C resulted in the degradation of phenolic acids by approximately 10% and 51%, respectively, when compared to vacuum drying at 50 °C. The content of phenolic acids after freeze and spray drying was approximately 30% lower in comparison to vacuum drying at 50 °C. Contrary to previous research on fruit powders [16,39], freeze drying did not ensure the highest retention of phenolic acids in juice powders. Previously, the addition of 15% maltodextrin to cranberry juice had a protective effect on chlorogenic acid during vacuum drying at up to 100 °C, as the content of the acid remained similar regardless of the temperature [39]. In the case of blackcurrant juice, vacuum drying resulted in the degradation of chlorogenic acid—its content was 2.2 times lower after vacuum drying at 90 °C when compared to vacuum drying at 50 °C, regardless of the carrier type and concentration. This finding indicates that the changes in individual polyphenols depended on the initial composition of the material subjected to drying. The type of carrier had a slight effect on the content of phenolic acids, whereas, similarly to flavonols, the content of phenolic acids was not affected by the carrier concentration.

### 2.2. Hydroxymethyl-l-Furfural

Hydroxymethyl-l-furfural was identified in all of the analyzed powders. Its content ranged between 3.9 μg/kg db, in freeze-dried powders containing 40% of maltodextrin, and 93.42 μg/kg db, in powders made by vacuum drying at 90 °C with the addition of inulin (Table 4). Similarly to plum juice powders with the addition of maltodextrin [16], the freeze-dried products contained HML regardless of the carrier type and concentration, which might have been due to the decomposition of carbohydrates present in the acidic environment of the juice [41]. On the other hand, the higher the temperature of the selected drying methods was, the higher the content of HMF was observed, which was, on average, 1.4, 2, 3.1, and 10 times higher for vacuum drying at 50 °C, spray drying, and vacuum drying at 70 °C and 90 °C, respectively, as compared to freeze drying. The carrier type (maltodextrin, inulin, and maltodextrin–inulin mixtures) and concentration were found to significantly affect the formation of HMF in blackcurrant juice powders.

In the case of vacuum drying at 50 and 70 °C and spray drying, the highest HMF content was noted for samples with the addition of maltodextrin, whereas in the case of vacuum drying at 90 °C, the highest level of HMF was observed when inulin was added. The HMF increase in the latter case is noteworthy, as it increased the average HMF content in samples containing inulin, so that it was about 33.3% higher than the average HMF content in powders with maltodextrin or inulin-maltodextrin mixtures regardless of the drying method and carrier concentration. Just like with plum juice powders made with the addition of maltodextrin [16], the formation of HMF decreased along with the increase in the concentration of the carrier added to the blackcurrant juice subjected to freeze drying and vacuum drying at 50 and 70 °C. A reverse correlation was observed in the case of vacuum drying at 90 °C and spray drying, where the highest temperatures were applied. The application of higher temperatures probably leads to the decomposition of carrier components, which may help the formation of HMF.

Previously, in the model system, the formation of hydroxymethyl-l-furfural was accelerated by the increased concentration of chlorogenic acid [42] under acidic conditions. As in the case of plum juice powders [16], no statistically significant correlation (*r* = −0.5075) between the content of chlorogenic acid and HMF was noted in the current study. This study proved that in fruit juices, which are more complex matrixes than artificial systems, the formation of Maillard reaction/caramelization products cannot be considered as involving only single compounds. It rather also includes interactions between the compounds and the influence of dominant constituents specific to the material subjected to drying. In the case of blackcurrant juice, a high, positive correlation was found between (+)-catechin and HMF (*r* = 0.9859). As little is said in the literature about a possible influence of (+)-catechin on the formation of HMF in blackcurrant juice powders, it could be assumed that HMF formation might be more connected with inulin conversion, and a decrease in the amount of inulin in the solution as a result of its conversion may improve the release of (+)-catechin from bounded structures. It should be mentioned that previous studies indicated a partial influence of ascorbic acid on the formation of HMF [16,43]. Since ascorbic acid was present close to the detection limit in selected samples (data not shown in this paper), a further study will be carried out to investigate the degradation of ascorbic acid in the products and whether it might significantly affect the formation of HMF. At the same time, it should be pointed out that inulin degradation had, most probably, a stronger influence on HMF formation during blackcurrant juice drying than ascorbic acid.

As hydroxymethyl-l-furfural is considered a quality indicator of processed foods, including fruit products [44,45], and its presence provides a clue about the food processing level, the quality of fruit powders should be assessed in a broader context, taking into account not only the process temperature or the carrier type and concentration, but also the chemical composition of the input material because it may determine the changes in the content of bioactive compounds, including the Maillard reaction/caramelization products.

### 2.3. Antioxidant Capacity

The antioxidant capacity of blackcurrant juice powders, measured in terms of the ability to scavenge the ABTS^•+^ radical cations and determined by FRAP method, was more strongly influenced by the carrier concentration than by the drying method and the carrier type (Table 4). Juice samples with the 30% carrier addition had, on average, 30% and 19.5% higher values of ABTS and FRAP, respectively, when compared to those with 40% of the carrier(s). Thus, the higher the share of a carrier or a carrier mixture in the blackcurrant juice, the lower the ability of the juice powder to scavenge the ABTS radicals and to reduce Fe^3+^ to Fe^2+^ [16]. When it comes to the applied drying methods, the powders vacuum dried at 90 °C showed the lowest ability to scavenge the ABTS^•+^ radical cations and the lowest FRAP values, which were 11% and 19% lower when compared to the highest values observed after spray drying and freeze drying. As the antioxidant capacity of the blackcurrant juice powders was the highest after freeze and spray drying, it can be concluded that the free-radical scavenging properties of the freeze- and spray-dried samples were strongly related to the content of compounds that are otherwise affected by the thermally induced changes during high-temperature drying. No statistically significant correlation was found between antioxidant capacity and the sum of identified polyphenolic compounds or HMF formed during drying. This was in agreement with the findings of the study on plum juice powders [16], where antioxidant capacity was found to be more strongly affected by the individual identified groups of polyphenolic compounds than by the compounds formed during thermal treatment.

### 2.4. Principal Component Analysis

**Figure 1:**
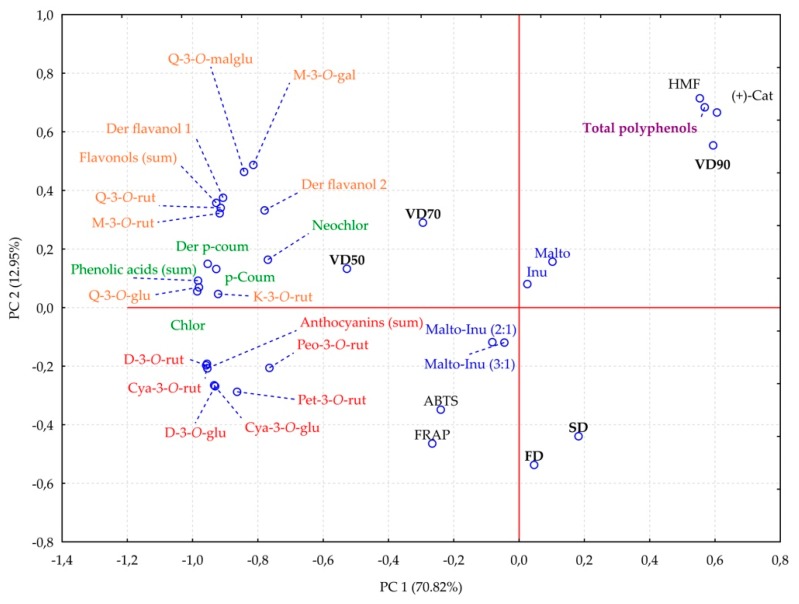
The principal component analysis (PCA) for drying methods (FD—freeze-drying; VD—vacuum drying at 50 °C, 70 °C, and 90 °C; SD—spray drying), carriers (Malto—maltodextrin; Inu—inulin; Malto-Inu (2:1 and 3:1)—Maltodextrin–inulin mixed in the proportions of 2:1 and 3:1), identified compounds (HMF—hydroxymethyl-l-furfural; (+)-cat—catechin; d-3-*O*-rut—delphinidin-3-*O*-rutinoside; Cya-3-*O*-rut—cyanidin-3-*O*-rutinoside; d-3-*O*-glu—delphinidin-3-*O*-glucoside; Cya-3-*O*-glu—cyanidin-3-*O*-glucoside; Pet-3-*O*-glu—petunidin-3-*O*-glucoside; Peo-3-*O*-rut—peonidin-3-*O*-rutinoside; Chlor—chlorogenic acid; *p*-Coum—*p*-coumaric acid; Der *p*-coum—derivative of *p*-coumaric acid; Neochlor—neochlorogenic acid; M-3-*O*-rut—myricetin-3-*O*-rutinoside; Q-3-*O*-rut—quercetin-3-*O*-rutinoside; Q-3-*O*-malglu—quercetin-3-*O*-malonylglucoside; M-3-*O*-gal—myricetin-3-*O*-galactoside; Der flavonol 1, 2—derivative of flavonol 1 and 2), and antioxidant capacity (ABTS—Trolox equivalent antioxidant capacity by ABTS^•+^; FRAP—Ferric Reducing Antioxidant Potential).

Principal component analysis was performed in order to establish the relationships between the drying methods, the carriers used, and the compounds identified in blackcurrant juice powders (Figure 1).

The PCA identified 4 main groups. The first group consisted of vacuum drying at 90 °C, HMF, total polyphenols, and (+)-catechin. In that case, the results strongly pointed to the influence of a high vacuum drying temperature on the formation of HMF and the release of (+)-catechin, which strongly influenced the sum of polyphenols identified in blackcurrant juice powders. The second group contained vacuum drying at 50 °C (VD50) and 70 °C (VD70), which were close to flavonols and phenolic acids, thus indicating a stronger influence of milder vacuum-drying conditions on the content of those constituents.

Antioxidant capacity measured by two different methods was more strongly influenced by anthocyanins than by the rest of the analyzed compounds. What is more, the antioxidant capacity values were more strongly affected by freeze and spray drying than by vacuum drying, regardless of the drying temperature. It can be stated that the carrier type has a lower impact on the compounds present in the blackcurrant juice, but carrier addition may not be disregarded in the assessment of the quality of the final products.

## 3. Materials and Methods

### 3.1. Material

The material used in the study was the fruit of the blackcurrant (*Ribes nigrum* L.) cultivar ‘Ruben’ bought from the experimental station ‘Przybroda’ (Rokietnica near Poznań), where it was grown according to the Integrated Fruit Production system (certificate of the Polish Main Inspectorate of Plant Health and Seed Inspection). After harvest, the fruit was frozen (–20 °C) and was kept in the frozen condition until the start of the study. Next, the fruit (20 kg) was thawed, steamed, and ground in a Thermomix (Wuppertal, Vorkwek, Germany) at 80 °C for 10 min. An enzyme Pectinex BE XXL (Novozyme A/S, Bagswaerd, Denmark) was then added at 50 °C for 2.5 h. Juice was extracted from the obtained pulp using a hydraulic press (extract content 12.4° ± 0.2° Brix). The juice was filtered and centrifuged (19,515× *g*, 5 min, 23 °C; MPW-380R, MPW - Med. Instruments, Warsaw, Poland). Afterwards, solutions with carrier concentrations of 30%, 35%, and 40% (*w*/*w*) were prepared. The carriers used to produce powders were maltodextrin DE (20–40) and inulin (Beneo-Orafti, Belgium) and their 2:1 and 3:1 mixtures (maltodextrin:inulin; *w*/*w*). The obtained juice-carrier compositions were then dried using selected drying methods.

### 3.2. Methods

#### 3.2.1. Drying methods

Blackcurrant juice with the addition of the carriers was freeze dried (FD) in an OE-950 freeze dryer (Labor, MIM, Budapest, Hungary) under reduced pressure (65 Pa) for 24 h. The temperature in the drying chamber was –60 °C, whereas the heating plate was heated up +25 °C. The freeze-dried sample was the control sample. Vacuum drying (VD) was carried out in a VACUCELL 111 ECO LINE vacuum dryer (MMM Medcenter Einrichtungen GmbH, Planegg, Germany) at 50, 70, and 90 °C under pressure of 0.1 mbar for 44, 20, and 16 h, respectively. The process duration and temperature were selected based on the study by Michalska et al. [39]. Spray drying (SD) was conducted using a B190 spray dryer (Buchi, Flawil, Switzerland) at an inlet temperature of 180 °C and an outlet temperature of 70 °C. Juice-carrier solutions had a temperature of 23 °C when entering the spray dryer, and the application speed was 400 mL/min.

Blackcurrant juice powders were obtained in duplicate using the above-described drying methods. The products were ground, vacuum sealed (PP–5.14, Tepro SA, Koszalin, Poland), and kept frozen at −20 °C before the analyses.

#### 3.2.2. Dry Basis

Dry basis was measured in duplicate at 80 °C for 20 h, according to the method by Figiel [46].

#### 3.2.3. Polyphenolic Content Measured by UPLC

Polyphenolic compounds were extracted from the juice powders in duplicate, according to the method described by Wojdyło et al. [47].

The qualitative and quantitative measurements of polyphenolic compounds were carried out using a UPLC Acquity system (Waters, Milford, MA, USA) with a PDA detector equipped with a binary pump system and a solvent manager. Analytes were separated on an Acquity BEH C18 column (100 mm × 1.7 μm, Waters, USA) at a flow velocity of 0.42 mL/min. A strong solvent (100% acetonitrile solution) and a weak solvent (10% acetonitrile–water solution) was run through the column. Polyphenolic compounds were separated by the gradient elution method using solvent A (4.5% formic acid) and solvent B (acetonitrile). Analytes were eluted as follows: 0–10 min linear gradient, 1–15% solvent B; 10–11.5 min linear gradient, 25–100% solvent B. The retention times of individual compounds were compared with standard retention times. The content of phenolic acids was determined at λ = 320 nm, flavonols at λ = 360 nm, and anthocyanins at λ = 520 nm. Standard curves were prepared based on chlorogenic acid, neochlorogenic acid, and cyanidin-3-*O*-glucoside and -rutinoside with a concentration of 0.05–5 g/L (r^2^ = 0.9998). The results were collected and analyzed using Empower 3 software. Measurements were made in duplicate for each sample and expressed in mg/kg of dry basis (db).

#### 3.2.4. Hydroxymethyl-l-Furfural Content

Hydroxymethyl-l-furfural content in the powders was measured with the Acquity UPLC system (Waters Corp., Milford, MA, USA) according to the procedure described by Gokmen and Senyuva [48]. The analysis was carried out in duplicate, and the results were expressed as an average μg/kg db.

#### 3.2.5. Antioxidant Capacity

The antioxidant capacity of extracts (0.005 g of sample in 1.7 mL of 80% aqueous methanol–hydrochloric acid mixture (1 mL/L)) was measured based on the ABTS^•+^ radical cation scavenging assay (Trolox equivalent antioxidant capacity (TEAC) ABTS) [49] and the FRAP assay [50] using a Synergy H1 spectrophotometer (BioTek Instruments Inc., Winnoski, VT, USA). The analysis was performed in duplicate, and results were presented in mmol Trolox equivalent/1 kg db.

#### 3.2.6. Statistical Analysis

Statistical analyses were performed using Statistica 10 (Statsoft, Tulsa, OK, USA). One-way analysis of variance ANOVA, the least significance HSD Tukey test, and a principal component analysis (PCA) were used to compare the results at *p* ≥ 0.05.

## 4. Conclusions

In total, 19 polyphenolic compounds were identified in blackcurrant juice powders, among which anthocyanins (6) were the dominant group followed by flavan-3-ol (1), flavonols (8), and phenolic acids (4). The sum of all identified polyphenols was significantly higher after vacuum drying at 90 °C when compared to the rest of the applied methods, which was connected with the thermally induced release of (+)-catechin during vacuum drying at 90 °C. Further, vacuum drying at 90 °C resulted in the highest formation of hydroxymethyl-l-furfural, as inulin was taking part in HMF formation. At the same time, inulin improved the retention of (+)-catechin because the decrease in inulin content during drying allowed a better release of (+)-catechin from polymerized structures. Taking the above into consideration, this study confirmed that the application of inulin resulted in a higher retention of the sum of identified polyphenolic compounds in blackcurrant juice powders when compared to the use of maltodextrin. The dominant group of polyphenolics present in blackcurrant juice powders was anthocyanins, with cyanidin- and delphinidin- 3-*O*-rutinosides being the major compounds. Vacuum drying significantly influenced the content of these compounds—the higher the drying temperature was, the stronger their degradation. Inulin was a better protectant of anthocyanins than maltodextrin, except during vacuum drying at 90 °C, which probably triggered the participation of inulin in the formation of HMF, thus limiting inulin’s ability to protect anthocyanins. As for flavonols and phenolic acids, vacuum drying at 50 °C resulted in their better retention compared to the rest of the drying methods used to prepare blackcurrant juice powders. The type of the added carrier affected only slightly, whereas the carrier concentration did not affect, the quantity of both groups of polyphenolic compounds. Taking the above into consideration, the quality of fruit juice powders should be considered by taking into account a broad spectrum of factors, including the initial composition of the material subjected to drying (as fruit juice is a complex matrix), the drying parameters, the carrier type and concentration, as well as the interactions between biomolecules themselves (including the resulting influence on antioxidant capacity) and between biomolecules and carriers (formation of HMF).

## Figures and Tables

**Table 1:** The content of anthocyanins (mg/kg db) identified in blackcurrant juice powders made with the addition of maltodextrin, inulin, and a mixture of maltodextrin–inulin using different drying methods (average ± SD; *n* = 3).

Carrier/Concentration		Anthocyanins						Flavan-3-ol
		Delphinidin-3-*O*-glucoside	Delphinidin-3-*O*-rutinoside	Cyanidin-3-*O*-glucoside	Cyanidin-3-*O*-rutinoside	Petunidin-3-*O*-rutinoside	Peonidin-3-*O*-rutinoside	(+)-catechin
	Freeze Drying							
**Maltodextrin**	40%	229.5 ± 5.4 *^ab^*	555.1 ± 5.9 *^ab^*	4.0 ± 0.1 *^bc^*	765.2 ± 9.6 *^ab^*	23.5 ± 4.0 *^a^*	16.6 ± 0.4 *^a^*	ND
	35%	252.1 ± 11.5 *^ab^*	620.3 ± 29.6 *^abc^*	4.7 ± 0.1 *^abc^*	888.4 ± 14.6 *^abcd^*	22.9 ± 2.9 *^a^*	17.7 ± 0.9 *^a^*	ND
	30%	279.6 ± 34.1 *^abc^*	718.4 ± 46.0 *^cdef^*	5.3 ± 0.4 *^a^*	1059.7 ± 19.0 *^cde^*	23.2 ± 1.2 *^a^*	23.3 ± 1.0 *^a^*	ND
**Maltodextrin:Inulin (2:1)**	40%	212.3 ± 3.6 *^a^*	527.0 ± 3.7 *^a^*	3.7 ± 0.0 *^b^*	740.8 ± 4.9 *^ab^*	23.6 ± 3.2 *^a^*	15.3 ± 8.8 *^a^*	ND
	35%	269.4 ± 1.5 *^abc^*	643.2 ± 3.3 *^abcde^*	4.4 ± 0.1 *^abc^*	878.9 ± 17.9 *^abcd^*	32.1 ± 0.5 *^abc^*	18.4 ± 0.1 *^a^*	ND
	30%	334.8 ± 8.9 *^ab^*	772.7 ± 15.9 *^def^*	5.5 ± 0.1 *^a^*	1084.8 ± 14.1 *^de^*	49.3 ± 0.5 *^d^*	26.8 ± 1.5 *^a^*	ND
**Maltodextrin:Inulin (3:1)**	40%	212.3 ± 2.5 *^a^*	522.2 ± 3.7 *^a^*	3.7 ± 0.0 *^b^*	717.6 ± 1.9 *^a^*	27.9 ± 0.5 *^ab^*	17.4 ± 0.9 *^a^*	ND
	35%	269.4 ± 7.0 *^abc^*	629.2 ± 12.5 *^abcd^*	4.5 ± 0.2 *^abc^*	868.4 ± 10.2 *^abc^*	29.4 ± 2.1 *^abc^*	16.6 ± 1.8 *^a^*	ND
	30%	341.1 ± 0.4 *^c^*	782.7 ± 16.9 *^ef^*	5.6 ± 0.2 *^a^*	1079.4 ± 39.2 *^cde^*	38.5 ± 4.8 *^c^*	24.5 ± 2.1 *^a^*	ND
**Inulin**	40%	234.7 ± 1.6 *^ab^*	537.0 ± 4.21 *^ab^*	4.0 ± 0.1 *^bc^*	736.4 ± 12.8 *^ab^*	22.5 ± 1.2 *^a^*	21.5 ± 0.2 *^a^*	ND
	35%	296.0 ± 13.4 *^bc^*	680.5 ± 15.0 *^bcdef^*	5.1 ± 0.1 *^ac^*	941.2 ± 8.7 *^bdec^*	32.7 ± 3.2 *^abc^*	27.8 ± 10.5 *^a^*	ND
	30%	333.5 ± 50.6 *^c^*	802.5 ± 116.2 *^f^*	5.5 ± 1.0 *^a^*	1140.2 ± 178.4 *^e^*	34.2 ± 2.7 *^bc^*	17.1 ± 0.2 *^a^*	ND
	Vacuum Drying 50 °C							
**Maltodextrin**	40%	321.2 ± 24.1 *^ab^*	873.1 ± 31.6 *^ef^*	5.5 ± 0.2 *^a^*	1205.2 ± 28.7 *^de^*	34.1 ± 0.3 *^abc^*	19.5 ± 1.5 *^abd^*	634.4 ± 11.0 *^g^*
	35%	244.0 ± 12.4 *^c^*	640.2 ± 15.6 *^c^*	4.3 ± 0.1 *^d^*	887.1 ± 22.5 *^c^*	29.9 ± 3.9 *^ac^*	16.8 ± 0.5 *^aa^*	433.1 ± 2.0 *^f^*
	30%	193.7 ± 2.6 *^c^*	512.5 ± 9.6 *^h^*	3.4 ± 0.1 *^e^*	697.0 ± 23.0 *^f^*	22.7 ± 1.2 *^c^*	14.0 ± 1.8 *^d^*	280.5 ± 9.9 *^e^*
**Maltodextrin:Inulin (2:1)**	40%	412.3 ± 8.2 *^efg^*	1021.5 ± 19.0 *^b^*	6.8 ± 0.2 *^bc^*	1426.7 ± 18.9 *^b^*	45.5 ± 4.7 *^bd^*	32.1 ± 0.8 *^ef^*	155.7 ± 14.7 *^ab^*
	35%	379.8 ± 19.3 *^def^*	940.0 ± 24.3 *^fg^*	6.6 ± 0.3 *^b^*	1308.8 ± 26.5 *^e^*	41.9 ± 0.2 *^abd^*	30.7 ± 4.6 *^ef^*	151.6 ± 4.92^*abc*^
	30%	243.5 ± 0.8 *^c^*	591.0 ± 2.3 *^c^*	4.4 ± 0.2 *^d^*	838.3 ± 15.6 *^c^*	32.0 ± 2.4 *^abc^*	21.2 ± 2.3 *^abcd^*	111.0 ± 17.4^*cd*^
**Maltodextrin:Inulin (3:1)**	40%	432.4 ± 11.0 *^g^*	1060.9 ± 23.7 *^b^*	7.4 ± 0.2 *^c^*	1492.1 ± 45 *^b^*	49.6 ± 7.9 *^d^*	33.4 ± 1.4 *^f^*	261.7 ± 12.9 *^e^*
	35%	328.8 ± 4.7 *^abd^*	788.7 ± 7.7 *^ad^*	5.6 ± 0.0 *^a^*	1087.6 ± 6.4 *^a^*	35.6 ± 1.4 *^abc^*	22.3 ± 1.1 *^abc^*	148.4 ± 7.0^*abc*^
	30%	311.5 ± 17.6 *^ab^*	759.4 ± 19.4 *^a^*	5.4 ± 0.4 *^a^*	1053.6 ± 29.6 *^a^*	35.1 ± 1.6 *^abc^*	20.9 ± 0.7 *^abcd^*	128.5 ± 12.8^*acd*^
**Inulin**	40%	362.8 ± 15.3 *^bde^*	854.4 ± 26.4 *^de^*	6.2 ± 0.4 *^ab^*	1197.6 ± 34.8 *^d^*	36.4 ± 3.9 *^abd^*	24.4 ± 2.1 *^abce^*	169.6 ± 0.5 *^ab^*
	35%	415.3 ± 12.7 *^fg^*	1009.5 ± 13.1 *^bg^*	7.0 ± 0.1 *^bc^*	1427.7 ± 10.4 *^b^*	35.4 ± 3.2 *^abc^*	25.9 ± 2.5 *^bcef^*	178.4 ± 15.8 *^b^*
	30%	307.3 ± 3.2 *^a^*	760.0 ± 5.7 *^a^*	5.4 ± 0.1 *^a^*	1073.3 ± 27.5 *^a^*	43.1 ± 2.0 *^abd^*	28.4 ± 0.4 *^cef^*	92.8 ± 3.0 *^d^*
	Vacuum Drying 70 °C							
**Maltodextrin**	40%	87.8 ± 2.2 *^b^*	310.4 ± 5.2 *^d^*	1.8 ± 0.0 *^c^*	433.6 ± 6.5 *^d^*	9.2 ± 0.6 *^e^*	8.0 ± 0.2 *^e^*	9994.1 ± 458.5 *^h^*
	35%	103.6 ± 6.1 *^b^*	341.9 ± 6.5 *^de^*	1.9 ± 0.2 *^cd^*	480.9 ± 18.2 *^de^*	14.1 ± 1.1 *^de^*	12.9 ± 2.9 *^de^*	7053.3 ± 6.3 *^g^*
	30%	135.4 ± 5.4 *^b^*	390.5 ± 3.8 *^e^*	2.6 ± 0.0 *^d^*	553.1 ± 0.9 *^e^*	24.8 ± 4.8 *^abd^*	14.4 ± 2.4 *^bde^*	4309.0 ± 221.6 *^d^*
**Maltodextrin:Inulin (2:1)**	40%	452.0 ± 10.5 *^d^*	1167.9 ± 28.6 *^h^*	7.7 ± 0.3 *^f^*	1651.4 ± 62.3 *^g^*	52.8 ± 2.2 *^f^*	43.4 ± 1.7 *^f^*	4636.9 ± 141.7 *^de^*
	35%	314.7 ± 17.4 *^a^*	799.7 ± 38.5 *^ab^*	5.5 ± 0.4 *^ab^*	1126.6 ± 39.4 *^bc^*	36.3 ± 0.4 *^abc^*	27.2 ± 7.0 *^a^*	2066.6 ± 82.6 *^a^*
	30%	324.2 ± 2.1 *^a^*	831.8 ± 1.7 *^ac^*	5.6 ± 0.0 *^ab^*	1176.0 ± 2.7 *^c^*	36.6 ± 1.4 *^bc^*	28.2 ± 1.6 *^a^*	1403.8 ± 0.4 *^b^*
**Maltodextrin:Inulin (3:1)**	40%	306.5 ± 1.4 *^a^*	787.7 ± 4.9 *^ab^*	5.5 ± 0.1 *^ab^*	1111.0 ± 10.8 *^abc^*	31.8 ± 2.4 *^abc^*	21.5 ± 0.5 *^abcd^*	5280.2 ± 75.7 *^f^*
	35%	299.3 ± 8.0 *^a^*	743.2 ± 10.0 *^bf^*	5.0 ± 0.1 *^a^*	1039.6 ± 4.1 *^ab^*	33.5 ± 4.6 *^abc^*	22.4 ± 0.6 *^abcd^*	3481.9 ± 22.1 *^c^*
	30%	207.9 ± 1.8 *^c^*	527.5 ± 4.2 *^g^*	3.7 ± 0.0 *^e^*	752.1 ± 12.1 *^f^*	24.6 ± 1.6 *^ad^*	16.4 ± 0.7 *^bcde^*	1995.2 ± 13.7 *^ab^*
**Inulin**	40%	341.6 ± 3.0 *^a^*	863.5 ± 0.7 *^c^*	6.0 ± 0.3 *^b^*	1204.6 ± 30.2 *^c^*	43.6 ± 5.8 *^cf^*	26.4 ± 1.6 *^ac^*	5031.8 ± 35.5 *^ef^*
	35%	338.2 ± 36.6 *^a^*	823.7 ± 12.2 *^ac^*	5.3 ± 0.2 *^ab^*	1062.2 ± 38.8 *^ab^*	34.5 ± 3.7 *^abc^*	23.1 ± 2.2 *^abc^*	3462.2 ± 0.0 *^c^*
	30%	291.7 ± 5.4 *^a^*	715.6 ± 0.6 *^f^*	5.0 ± 0.0 *^a^*	1008.5 ± 2.8 *^a^*	30.5 ± 0.4 *^ab^*	23.9 ± 1.3 *^abc^*	2149.0 ± 1.2 *^a^*
	Vacuum Drying 90 °C							
**Maltodextrin**	40%	167.7 ± 2.4 *^f^*	434.9 ± 7.5 *^c^*	2.9 ± 0.1 *^ef^*	605.7 ± 12.5 *^c^*	22.5 ± 4.6 *^abc^*	16.7 ± 2.3 *^abc^*	9759.3 ± 175.6 *^a^*
	35%	192.4 ± 5.9 *^g^*	507.1± 8.3 *^g^*	3.4 ± 0.2 *^fg^*	716.7 ± 5.8 *^g^*	25.1 ± 3.8 *^ab^*	22.5 ± 1.3 *^ac^*	11393.2 ± 167.2 *^a^*
	30%	216.6 ± 5.0 *^h^*	583.7 ± 1.3 *^h^*	3.8 ± 0.1 *^g^*	816.3 ± 3.6 *^h^*	28.4 ± 6.0 *^b^*	24.7 ± 0.9 *^a^*	17945.7 ± 2.1 *^f^*
**Maltodextrin:Inulin (2:1)**	40%	95.3 ± 16.5 *^c^*	300.5 ± 6.3 *^e^*	1.7 ± 0.4 *^a^*	420.2 ± 9.2 *^e^*	11.7 ± 2.0 *^cd^*	12.8 ± 0.2 *^b^*	22266.3 ± 135.9 *^b^*
	35%	116.0 ± 3.6 *^cd^*	346.8 ± 10.6 *^a^*	2.2 ± 0.0 *^ab^*	484.2 ± 3.9 *^a^*	20.7 ± 1.4 *^abc^*	18.0 ± 2.0 *^abc^*	30796.3 ± 832.1 *^c^*
	30%	117.3 ± 1.7 *^cd^*	360.6 ± 1.5 *^a^*	2.1 ± 0.1 *^ab^*	506.1 ± 1.1 *^a^*	23.0 ± 2.0 *^abc^*	17.7 ± 4.2 *^abc^*	40205.2 ± 218.7*^g^*
**Maltodextrin:Inulin (3:1)**	40%	124.4 ± 3.9 *^de^*	354.0 ± 2.7 *^a^*	2.3 ± 0.0 *^abd^*	497.3 ± 4.0 *^a^*	15.8 ± 3.3 *^ac^*	13.0 ± 0.1 *^bc^*	15327.5 ± 140.0 *^e^*
	35%	140.0 ± 0.2 *^ef^*	408.3 ± 13.6 *^f^*	2.5 ± 0.0 *^bde^*	583.8 ± 8.6 *^c^*	17.3 ± 0.5 *^abc^*	21.4 ± 1.0 *^abc^*	21339.8 ± 790.5 *^b^*
	30%	157.3 ± 2.4 *^fg^*	452.4 ± 0.6 *^c^*	2.7 ± 0.1 *^de^*	634.4 ± 12.1 *^f^*	24.2 ± 5.1 *^ab^*	23.0 ± 6.3 *^a^*	32298.7 ± 310.4 *^c^*
**Inulin**	40%	22.0 ± 2.0 *^a^*	70.3 ± 2.2 *^d^*	0.3 ± 0.0 *^c^*	99.4 ± 1.4 *^d^*	ND	ND	43485.5 ± 43.5 *^h^*
	35%	9.9 ± 2.1 *^a^*	27.8 ± 0.8 *^b^*	0.1 ± 0.0 *^c^*	46.8 ± 2.6 *^b^*	ND	ND	54606.9 ± 1041.4 *^d^*
	30%	6.8 ± 1.6 *^a^*	26.7 ± 3.7 *^b^*	0.2 ± 0.1 *^c^*	42.3 ± 0.4 *^b^*	ND	ND	55693.0 ± 223.0*^d^*
	Spray Drying							
**Maltodextrin**	40%	135.7 ± 7.4 *^f^*	350.1 ± 12.1 *^f^*	2.3 ± 0.0 *^d^*	465.8 ± 6.3 *^f^*	21.3 ± 2.6 *^a^*	14.6 ± 2.4 *^a^*	1194.4 ± 25.9 *^a^*
	35%	194.4 ± 12.7 *^ab^*	489.8 ± 17.5 *^a^*	3.2 ± 0.3 *^ade^*	657.4 ± 23.3 *^ab^*	26.4 ± 4.1 *^ab^*	19.3 ± 0.2 *^ab^*	1679.4 ± 30.1 *^b^*
	30%	230.6 ± 2.5 *^bcd^*	600.7 ± 8.2 *^cf^*	4.1 ± 0.2 *^abc^*	833.7 ± 7.9 *^dg^*	28.0 ± 3.8 *^ab^*	29.7 ± 4.7 *^b^*	5546.5 ± 182.4 *^f^*
**Maltodextrin:Inulin (2:1)**	40%	1355 ± 4.3 *^f^*	340.8 ± 13.8 *^f^*	2.4 ± 0.1 *^de^*	478.0 ± 3.0 *^f^*	19.7 ± 6.2 *^a^*	14.3 ± 3.6 *^a^*	709.2 ± 19.3 *^d^*
	35%	203.7 ± 6.2 *^abc^*	522.0 ± 9.6 *^ab^*	3.5 ± 0.0 *^ac^*	731.3 ± 3.0 *^bc^*	22.9 ± 1.5 *^a^*	13.9 ± 5.5 *^a^*	992.6 ± 58.4 *^a^*
	30%	283.3 ± 22.1 *^e^*	708.0 ± 34.1 *^d^*	4.8 ± 0.7 *^b^*	986.4 ± 35.1 *^e^*	30.0 ± 3.9 *^ab^*	18.5 ± 5.3 *^ab^*	1785.0 ± 8.0 *^bc^*
**Maltodextrin:Inulin (3:1)**	40%	188.3 ± 5.7 *^a^*	475.2 ± 23.0 *^a^*	3.3 ± 0.1 *^ae^*	662.2 ± 35.1 *^ab^*	20.7 ± 3.1 *^a^*	20.7 ± 1.2 *^ab^*	1590.5 ± 78.6 *^b^*
	35%	231.0 ± 5.6 *^bcd^*	580.8 ± 2.8 *^bc^*	3.9 ± 0.2 *^abc^*	794.8 ± 4.2 *^cd^*	28.1 ± 1.2 *^ab^*	19.8 ± 1.6 *^ab^*	1973.3 ± 62.0 *^c^*
	30%	278.4 ± 4.4 *^e^*	693.2 ± 2.1 *^d^*	4.7 ± 0.1 *^b^*	955.0 ± 9.7 *^e^*	31.2 ± 0.5 *^ab^*	18.6 ± 0.7 *^ab^*	1654.7 ± 24.8 *^b^*
**Inulin**	40%	189.4 ± 9.2 *^a^*	462.6 ± 19.3 *^a^*	3.2 ± 0.2 *^ade^*	630.3 ± 18.1 *^a^*	26.3 ± 2.7 *^ab^*	18.00 ± 2.8 *^ab^*	1122.1 ± 38.0 *^a^*
	35%	232.6 ± 11.2 *^cd^*	581.9 ± 28.0 *^bc^*	4.0 ± 0.1 *^abc^*	798.8 ± 49.0 *^cd^*	31.2 ± 1.5 *^ab^*	21.9 ± 1.6 *^ab^*	1138.3 ± 7.8 *^a^*
	30%	256.6 ± 6.8 *^de^*	661.3 ± 15.9 *^df^*	4.4 ± 0.1 *^bc^*	908.5 ± 15.8 *^eg^*	38.5 ± 2.5 *^b^*	31.5 ± 5.5 *^b^*	3195.5 ± 23.2 *^e^*

ND—not detected; a, b, c, d, e, f, g, h—different letters within groups stand for different drying methods, i.e., freeze drying, vacuum drying at 50 °C, 70 °C, and 90 °C, and spray drying, and they indicate significant differences (ANOVA, HSD Tukey, *p* ≥ 0.05).

**Table 2:** The content of flavonols (mg/kg db) identified in blackcurrant juice powders made with the addition maltodextrin, inulin, and a mixture of maltodextrin–inulin using different drying methods (average ± SD; *n* = 3).

Carrier/Concentration		Myr-3-*O*-rutinoside	Myr-3-*O*-galactoside	Q-3-*O*-rutinoside	Q-3-*O*-glucoside	Q-3-*O*-malonylglucoside	Der of Flavonol 1	K-3-*O*-rutinoside	Der of Flavonols
	Freeze Drying								
**Maltodextrin**	40%	20.4 ± 1.5 *^a^*	40.1 ± 5.6 *^a^*	71.6 ± 1.6 *^ad^*	20.3 ± 0.5 *^a^*	25.1 ± 1.7 *^ab^*	33.0 ± 0.2 *^abc^*	12.3 ± 1.0 *^bc^*	9.3 ± 1.0 *^ac^*
	35%	26.8 ± 1.2 *^d^*	46.5 ± 2.0 *^abc^*	82.8 ± 0.8 *^b^*	22.5 ± 0.3 *^abc^*	26.6 ± 1.5 *^ab^*	38.5 ± 0.9 *^ad^*	13.4 ± 0.9 *^abc^*	10.3 ± 0.7 *^acd^*
	30%	30.8 ± 0.7 *^c^*	54.0 ± 1.3 *^bcd^*	96.4 ± 0.2 *^c^*	30.2 ± 2.0 *^d^*	34.8 ± 1.0 *^c^*	47.4 ± 2.4 *^f^*	19.2 ± 1.2 *^ad^*	16.1 ± 0.2 *^ef^*
**Maltodextrin:Inulin (2:1)**	40%	21.5 ± 0.6 *^ab^*	42.8 ± 1.3 *^ab^*	69.0 ± 4.3 *^ad^*	21.1 ± 0.9 *^ab^*	24.4 ± 0.4 *^ab^*	31.9 ± 0.8 *^abc^*	16.9 ± 0.1 *^abcd^*	9.0 ± 0.4 *^a^*
	35%	25.1 ± 0.9 *^bd^*	44.9 ± 0.2 *^ab^*	82.3 ± 5.3 *^b^*	23.9 ± 0.2 *^bc^*	28.2 ± 2.7 *^abd^*	37.5 ± 1.8 *^acd^*	18.2 ± 1.3 *^abcd^*	12.0 ± 0.5 *^bcd^*
	30%	33.8 ± 0.9 *^c^*	59.8 ± 2.6 *^d^*	99.5 ± 1.0 *^c^*	31.4 ± 0.1 *^d^*	36.3 ± 0.6	46.0 ± 2.0 *^ef^*	23.1 ± 0.8 *^d^*	18.6 ± 1.4 *^f^*
**Maltodextrin:Inulin (3:1)**	40%	21.6 ± 1.1 *^ab^*	40.0 ± 0.8 *^a^*	65.4 ± 2.8 *^a^*	19.7 ± 0.3 *^a^*	25.1 ± 2.1 *^ab^*	30.8 ± 0.7 *^bc^*	11.7 ± 0.7 *^b^*	13.4 ± 1.0 *^be^*
	35%	22.6 ± 0.4 *^ab^*	45.7 ± 3.0 *^ab^*	78.3 ± 0.2 *^bd^*	25.4 ± 0.8 *^c^*	33.5 ± 2.3 *^cde^*	36.0 ± 2.6 *^abc^*	19.1 ± 1.1 *^ad^*	14.9 ± 0.6 *^be^*
	30%	31.4 ± 0.9 *^c^*	57.5 ± 4.1 *^cd^*	100.1 ± 3.5 *^c^*	29.4 ± 0.5 *^d^*	34.4 ± 0.3 *^ce^*	43.8 ± 1.5 *^def^*	18.8 ± 1.4 *^acd^*	12.6 ± 0.3 *^bd^*
**Inulin**	40%	21.9 ± 0.6 *^ab^*	39.1 ± 1.0 *^a^*	66.7 ± 1.5 *^a^*	20.5 ± 0.7 *^a^*	22.5 ± 0.7 *^a^*	30.0 ± 0.7 *^b^*	13.0 ± 0.0 *^abc^*	10.1 ± 0.3 *^acd^*
	35%	26.7 ± 0.5 *^d^*	47.0 ± 0.4 *^abc^*	82.6 ± 3.2 *^b^*	24.1 ± 0.4 *^bc^*	28.7 ± 1.1 *^bde^*	39.4 ± 0.6 *^ade^*	13.2 ± 0.1 *^abc^*	8.7 ± 0.7 *^a^*
	30%	32.2 ± 1.6 *^c^*	58.0 ± 5.7 *^cd^*	100.8 ± 0.9 *^c^*	30.2 ± 0.7 *^d^*	34.0 ± 1.0 *^cde^*	46.3 ± 4.2 *^ef^*	20.2 ± 4.9 *^d^*	13.6 ± 1.0 *^be^*
	Vacuum Drying 50 °C								
**Maltodextrin**	40%	50.3 ± 3.1 *^ef^*	96.6 ± 8.5 *^bc^*	175.4 ± 1.9 *^g^*	45.2 ± 3.9 *^e^*	64.4 ± 1.9 *^f^*	77.5 ± 1.2 *^b^*	29.2 ± 2.7 *^acd^*	32.5 ± 0.8 *^ab^*
	35%	34.1 ± 0.2 *^abc^*	67.1 ± 1.3 *^ae^*	113.7 ± 7.4 *^bce^*	29.6 ± 0.8 *^ab^*	41.0 ± 2.8 *^ac^*	52.5 ± 4.8 *^a^*	21.0 ± 1.4 *^ab^*	25.0 ± 1.3 *^de^*
	30%	27.6 ± 1.8 *^a^*	50.1 ± 2.7 *^de^*	83.4 ± 2.0 *^a^*	25.8 ± 2.3 *^a^*	30.9 ± 0.0 *^b^*	42.1 ± 1.0 *^e^*	19.4 ± 0.9 *^ab^*	20.9 ± 0.2 *^cd^*
**Maltodextrin:Inulin (2:1)**	40%	45.6 ± 1.6 *^def^*	82.7 ± 3.6 *^c^*	145.7 ± 2.5 *^d^*	41.1 ± 0.3 *^def^*	53.0 ± 2.5 *^de^*	69.2 ± 0.4 *^bd^*	32.4 ± 1.1 *^cd^*	33.0 ± 2.7 *^ab^*
	35%	42.6 ± 1.8 *^cde^*	81.1 ± 1.1 *^ab^*	131.8 ± 1.6 *^def^*	39.1 ± 0.5 *^cdef^*	47.5 ± 1.0 *^ad^*	65.7 ± 3.3 *^cd^*	29.5 ± 2.8 *^acd^*	30.7 ± 1.7 *^abe^*
	30%	28.9 ± 0.7 *^a^*	49.2 ± 1.5 *^d^*	86.8 ± 0.3 *^a^*	26.1 ± 2.0 *^a^*	31.2 ± 0.3 *^b^*	38.4 ± 1.2 *^e^*	20.6 ± 0.9 *^ab^*	14.2 ± 4.2 *^f^*
**Maltodextrin:Inulin (3:1)**	40%	52.7 ± 1.7 *^f^*	110.6 ± 3.2 *^ab^*	149.1 ± 3.1 *^d^*	47.2 ± 0.6 *^e^*	56.3 ± 3.1 *^e^*	75.1 ± 0.1 *^b^*	33.1 ± 3.0 *^d^*	35.4 ± 1.4 *^b^*
	35%	37.8 ± 0.7 *^bcd^*	82.3 ± 4.3 *^ab^*	105.6 ± 2.4 *^bc^*	33.3 ± 3.3 *^abce^*	44.9 ± 1.8 *^ac^*	56.2 ± 0.1 *^a^*	20.8 ± 4.3 *^ab^*	16.7 ± 0.3 *^cf^*
	30%	34.2 ± 4.0 *^abc^*	75.8 ± 7.0 *^a^*	96.6 ± 5.2 *^ab^*	32.5 ± 0.5 *^abc^*	37.4 ± 1.1 *^bc^*	51.4 ± 0.4 *^a^*	22.6 ± 1.2 *^abc^*	18.3 ± 0.7 *^ef^*
**Inulin**	40%	40.3 ± 2.6 *^bcd^*	80.3 ± 4.4 *^ab^*	118.8 ± 2.0 *^cef^*	36.6 ± 3.8 *^bcef^*	46.8 ± 1.8 *^ad^*	60.1 ± 2.5 *^ac^*	26.4 ± 0.2 *^abcd^*	30.8 ± 0.2 *^abe^*
	35%	46.1 ± 3.5 *^def^*	96.6 ± 1.5 *^bc^*	133.3 ± 11.8 *^df^*	44.0 ± 0.8 *^ef^*	47.9 ± 3.0 *^ad^*	68.9 ± 2.9 *^bcd^*	28.6 ± 5.7 *^acd^*	26.4 ± 0.0 *^ade^*
	30%	33.6 ± 0.2 *^ab^*	74.9 ± 5.1 *^a^*	101.3 ± 0.9 *^abc^*	32.0 ± 1.0 *^abc^*	38.0 ± 0.7 *^bc^*	51.3 ± 2.9 *^a^*	17.6 ± 0.4 *^b^*	22.0 ± 1.1 *^cd^*
	Vacuum Drying 70 °C								
**Maltodextrin**	40%	39.8 ± 0.4 *^abc^*	93.6 ± 1.0 *^b^*	123.9 ± 0.5 *^bd^*	29.4 ± 1.2 *^bd^*	53.9 ± 6.1 *^def^*	62.0 ± 3.1 *^a^*	17.9 ± 2.2 *^acd^*	19.5 ± 1.2 *^ab^*
	35%	36.6 ± 1.7 *^ab^*	76.9 ± 5.5 *^a^*	101.7 ± 2.6 *^a^*	24.8 ± 0.6 *^cd^*	39.0 ± 2.0 *^abc^*	50.0 ± 1.6 *^bd^*	14.6 ± 0.9 *^d^*	18.2 ± 2.5 *^abc^*
	30%	25.4 ± 2.2 *^d^*	59.4 ± 4.8 *^c^*	78.8 ± 2.6 *^e^*	20.8 ± 0.3 *^c^*	30.7 ± 3.5 *^c^*	39.4 ± 0.4 *^cd^*	12.5 ± 1.5 *^d^*	18.6 ± 0.6 *^abc^*
**Maltodextrin:Inulin (2:1)**	40%	51.9 ± 1.0 *^e^*	115.8 ± 2.2 *^d^*	162.2 ± 1.7 *^f^*	44.6 ± 0.5 *^e^*	61.7 ± 1.3	74.7 ± 0.7 *^e^*	32.9 ± 2.6 *^e^*	20.2 ± 0.4 *^ab^*
	35%	37.4 ± 1.1 *^ab^*	75.6 ± 2.8 *^a^*	109.8 ± 3.5 *^ac^*	33.1 ± 1.2 *^ab^*	43.6 ± 5.8 *^abd^*	53.2 ± 3.7 *^ab^*	26.1 ± 2.7 *^be^*	17.2 ± 0.4 *^abc^*
	30%	35.9 ± 1.6 *^a^*	77.1 ± 2.5 *^a^*	110.4 ± 3.9 *^ac^*	33.6 ± 1.1 *^ab^*	44.9 ± 2.3 *^abd^*	56.6 ± 2.5 *^ab^*	22.0 ± 0.6 *^abc^*	15.8 ± 1.6 *^ac^*
**Maltodextrin:Inulin (3:1)**	40%	42.1 ± 1.7 *^bc^*	86.5 ± 6.8 *^ab^*	123.2 ± 1.4 *^bd^*	34.4 ± 1.4 *^ab^*	47.6 ± 0.1 *^abde^*	57.1 ± 1.8 *^ab^*	24.3 ± 2.6 *^ab^*	17.2 ± 0.1 *^abc^*
	35%	36.8 ± 2.8 *^ab^*	78.9 ± 6.4 *^ab^*	113.9 ± 0.0 *^cd^*	31.0 ± 0.1 *^ab^*	38.2 ± 0.6 *^ac^*	55.7 ± 3.9 *^ab^*	22.5 ± 2.5 *^abc^*	20.8 ± 3.4 *^ab^*
	30%	27.2 ± 2.3 *^d^*	56.4 ± 3.9 *^c^*	80.1 ± 2.8 *^e^*	24.0 ± 1.7 *^cd^*	31.5 ± 0.2 *^c^*	37.8 ± 1.2 *^c^*	15.9 ± 0.3 *^cd^*	12.5 ± 1.4 *^c^*
**Inulin**	40%	43.6 ± 0.2 *^c^*	89.3 ± 0.4 *^ab^*	128.3 ± 0.0 *^b^*	36.6 ± 0.0 *^a^*	50.3 ± 1.3 *^bdef^*	62.3 ± 3.0 *^a^*	22.0 ± 0.8 *^abc^*	15.0 ± 1.1 *^ac^*
	35%	44.7 ± 0.0 *^c^*	86.5 ± 1.0 *^ab^*	128.9 ± 6.9 *^b^*	36.8 ± 1.8 *^a^*	59.0 ± 0.0 *^ef^*	59.0 ± 4.8 *^ab^*	22.5 ± 1.3 *^abc^*	23.1 ± 0.8 *^b^*
	30%	34.6 ± 0.4 *^a^*	77.1 ± 1.4 *^a^*	100.1 ± 2.8 *^a^*	33.4 ± 3.9 *^ab^*	41.9 ± 3.7 *^abc^*	53.4 ± 1.3 *^ab^*	24.8 ± 1.0 *^ab^*	23.5 ± 2.9 *^b^*
	Vacuum Drying 90 °C								
**Maltodextrin**	40%	22.3 ± 0.6 *^abc^*	50.0 ± 3.6 *^abcd^*	64.9 ± 4.8 *^ace^*	17.8 ± 1.2 *^ab^*	24.9 ± 2.0 *^abc^*	32.7 ± 1.1 *^ae^*	13.4 ± 1.6 *^abc^*	9.3 ± 0.2 *^ab^*
	35%	24.5 ± 1.6 *^ad^*	55.2 ± 0.4 *^cde^*	74.0 ± 2.2 *^de^*	22.2 ± 2.5 *^ab^*	31.0 ± 2.6 *^abcd^*	39.4 ± 1.7 *^ce^*	15.7 ± 0.1 *^bc^*	14.2 ± 2.3 *^ac^*
	30%	27.0 ± 0.5 *^d^*	66.5 ± 2.0 *^f^*	89.6 ± 6.2 *^f^*	24.5 ± 2.9 *^b^*	35.0 ± 2.8 *^cd^*	44.5 ± 2.1 *^c^*	16.6 ± 1.1 *^c^*	16.5 ± 2.6 *^c^*
**Maltodextrin:Inulin (2:1)**	40%	19.4 ± 0.6 *^bc^*	45.6 ± 1.7 *^abc^*	57.5 ± 1.4 *^ab^*	15.4 ± 0.7 *^acd^*	21.9 ± 2.1 *^a^*	29.3 ± 3.8 *^ab^*	14.9 ± 1.0 *^bc^*	10.4 ± 1.6 *^abc^*
	35%	23.6 ± 0.6 *^acd^*	54.3 ± 6.0 *^bcde^*	70.5 ± 1.1 *^cde^*	19.4 ± 4.5 *^ab^*	31.2 ± 6.3 *^abcd^*	33.4 ± 1.4 *^ae^*	10.6 ± 1.7 *^abd^*	12.1 ± 1.5 *^abc^*
	30%	24.9 ± 0.3 *^ad^*	62.3 ± 0.0 *^ef^*	77.8 ± 1.5 *^d^*	18.8 ± 2.0 *^ab^*	36.7 ± 0.7 *^d^*	41.1 ± 1.3 *^cd^*	13.2 ± 2.5 *^abc^*	14.0 ± 2.8 *^ac^*
**Maltodextrin:Inulin (3:1)**	40%	20.8 ± 1.4 *^abc^*	44.9 ± 1.1 *^ab^*	57.6 ± 1.1 *^ab^*	16.7 ± 0.6 *^ad^*	24.5 ± 1.0 *^ab^*	30.8 ± 0.6 *^ab^*	11.0 ± 0.7 *^abcd^*	10.0 ± 1.0 *^ab^*
	35%	24.3 ± 0.9 *^ad^*	56.2 ± 1.4 *^de^*	62.8 ± 0.8 *^ac^*	21.4 ± 1.0 *^ab^*	28.9 ± 1.3 *^abcd^*	46.7 ± 0.8 *^d^*	12.6 ± 0.7 *^abc^*	12.5 ± 0.63 *^abc^*
	30%	28.2 ± 0.0 *^d^*	69.3 ± 3.8 *^f^*	78.3 ± 1.4 *^d^*	22.2 ± 1.5 *^ab^*	32.6 ± 1.8 *^bcd^*	43.1 ± 0.6 *^cd^*	12.6 ± 1.8 *^abc^*	12.9 ± 1.8 *^abc^*
**Inulin**	40%	17.8 ± 0.1 *^a^*	43.6 ± 0.4 *^a^*	49.4 ± 1.3 *^b^*	9.5 ± 0.2 *^cd^*	25.1 ± 0.6 *^abc^*	24.2 ± 0.8 *^b^*	8.5 ± 1.2 *^ad^*	8.5 ± 0.2 *^ab^*
	35%	19.1 ± 1.4 *^bc^*	45.0 ± 0.4 *^ab^*	55.6 ± 0.9 *^ab^*	9.6 ± 0.5 *^cd^*	24.6 ± 3.6 *^abc^*	28.7 ± 1.2 *^ab^*	8.7 ± 2.3 *^ad^*	8.8 ± 0.4 *^ab^*
	30%	21.7 ± 2.8 *^abc^*	55.0 ± 0.1 *^cde^*	62.9 ± 0.6 *^ac^*	8.7 ± 0.1 *^c^*	26.9 ± 0.2 *^abcd^*	30.9 ± 2.7 *^ab^*	6.1 ± 1.1 *^d^*	7.7 ± 0.4 *^b^*
	Spray Drying								
**Maltodextrin**	40%	21.9 ± 3.9 *^ad^*	47.7 ± 0.5 *^ad^*	55.9 ± 2.6 *^a^*	18.1 ± 2.5 *^ad^*	23.8 ± 0.0 *^abd^*	28.8 ± 1.7 *^a^*	13.2 ± 0.3 *^ab^*	9.1 ± 0.2 *^ab^*
	35%	29.5 ± 1.9 *^abc^*	59.9 ± 2.3 *^be^*	73.0 ± 5.1 *^be^*	26.3 ± 1.7 *^abc^*	27.9 ± 3.2 *^abc^*	36.9 ± 1.8 *^be^*	18.4 ± 2.2 *^abcd^*	9.0 ± 0.5 *^ab^*
	30%	31.1 ± 0.5 *^abc^*	67.8 ± 1.1 *^bc^*	82.1 ± 2.4 *^bc^*	26.4 ± 2.1 *^abc^*	31.6 ± 4.0 *^bce^*	42.4 ± 1.0 *^bcd^*	16.0 ± 0.8 *^abcd^*	12.7 ± 1.3 *^a^*
**Maltodextrin:Inulin (2:1)**	40%	13.5 ± 1.0 *^d^*	30.9 ± 0.4 *^f^*	38.0 ± 1.1 *^f^*	14.9 ± 0.4 *^d^*	17.3 ± 1.4 *^d^*	20.9 ± 2.0 *^f^*	13.6 ± 0.7 *^abc^*	6.1 ± 0.5 *^b^*
	35%	23.1 ± 3.2 *^ad^*	49.4 ± 0.4 *^ad^*	57.4 ± 1.2 *^a^*	20.2 ± 2.3 *^ad^*	24.7 ± 0.3 *^abd^*	30.0 ± 0.7 *^a^*	12.1 ± 1.3 *^a^*	11.7 ± 2.6 *^abc^*
	30%	30.0 ± 0.7 *^abc^*	67.8 ± 0.9 *^bc^*	82.8 ± 0.7*^bcc^*	28.9 ± 0.8 *^bc^*	30.4 ± 1.27 *^abce^*	48.2 ± 2.0 *^d^*	20.3 ± 1.2 *^bcd^*	14.8 ± 0.6 *^a^*
**Maltodextrin:Inulin (3:1)**	40%	23.0 ± 4.0 *^ad^*	47.1 ± 3.7 *^a^*	61.1 ± 2.7 *^ad^*	19.5 ± 2.5 *^ad^*	22.7 ± 3.36 *^ad^*	28.4 ± 2.0 *^a^*	13.1 ± 0.6 *^ab^*	14.7 ± 3.1 *^a^*
	35%	26.6 ± 2.6 *^abc^*	56.2 ± 0.2 *^de^*	69.6 ± 1.7 *^de^*	23.6 ± 1.0 *^ab^*	29.1 ± 0.5 *^abc^*	36.6 ± 0.9 *^be^*	18.0 ± 0.4 *^abcd^*	11.7 ± 0.2 *^ab^*
	30%	32.9 ± 3.7 *^bc^*	68.9 ± 5.2 *^abc^*	88.4 ± 2.1 *^c^*	31.3 ± 3.0 *^bc^*	33.2 ± 0.6 *^ce^*	44.8 ± 0.0 *^cd^*	20.6 ± 4.0 *^cd^*	11.9 ± 2.3 *^abc^*
**Inulin**	40%	23.4 ± 1.3 *^ab^*	45.2 ± 0.9 *^a^*	58.2 ± 4.1 *^a^*	20.3 ± 0.8 *^ad^*	27.8 ± 2.6 *^abc^*	33.4 ± 2.4 *^ae^*	13.6 ± 0.9 *^abc^*	10.4 ± 1.9 *^ab^*
	35%	25.5 ± 2.0 *^abc^*	58.7 ± 1.4 *^e^*	71.7 ± 3.3 *^bde^*	26.0 ± 1.3 *^abc^*	27.6 ± 0.3 *^abc^*	41.4 ± 0.9 *^bc^*	20.8 ± 0.7 *^cd^*	9.8 ± 0.0 *^ab^*
	30%	34.6 ± 0.4 *^c^*	68.8 ± 1.3 *^bc^*	89.4 ± 4.0 *^c^*	32.5 ± 4.3 *^c^*	38.3 ± 1.6 *^e^*	45.6 ± 0.6 *^cd^*	21.3 ± 3.7 *^d^*	11.1 ± 1.2 *^ab^*

Myr—myricetin; Q—quercetin; Der—derivative; K—kaempferol; a, b, c, d, e, f, g —different letters within groups stand for different drying methods, i.e., freeze drying, vacuum drying at 50 °C, 70 °C, and 90 °C, and spray drying, and they indicate significant differences (ANOVA, HSD Tukey, *p* ≥ 0.05).

**Table 3:** The content of phenolic acids (mg/kg db) identified in blackcurrant juice powders made with the addition maltodextrin, inulin, and a mixture of maltodextrin–inulin using different drying methods (average ± SD; *n* = 3).

Carrier/Concentration		Neochlorogenic Acid	*p*-Coumaric Acid	Chlorogenic Acid	Derivative of *p*-Coumaric
	Freeze Drying				
**Maltodextrin**	40%	5.5 ± 0.8 *^ad^*	6.2 ± 0.4 *^c^*	44.5 ± 2.2 *^b^*	19.2 ± 1.6 *^abc^*
	35%	5.0 ± 0.2 *^abcd^*	9.0 ± 0.5 *^abcd^*	54.7 ± 0.4 *^acd^*	22.1 ± 0.3 *^bcde^*
	30%	5.5 ± 0.3 *^ad^*	10.6 ± 0.7 *^b^*	65.9 ± 0.6 *^fg^*	24.3 ± 0.7 *^ef^*
**Maltodextrin:Inulin (2:1)**	40%	4.2 ± 0.5 *^bcd^*	7.4 ± 0.2 *^acd^*	45.8 ± 0.3 *^ab^*	18.4 ± 1.2 *^ab^*
	35%	4.2 ± 0.0 *^bc^*	8.6 ± 0.2 *^abcd^*	55.4 ± 1.1 *^acde^*	19.7 ± 0.3 *^abcd^*
	30%	6.0 ± 0.2 *^a^*	11.3 ± 0.5 *^b^*	69.7 ± 1.4	28.3 ± 0.9 *^g^*
**Maltodextrin:Inulin (3:1)**	40%	3.7 ± 0.2 *^b^*	7.1 ± 0.5 *^ac^*	45.7 ± 1.3 *^ab^*	19.1 ± 0.0 *^ab^*
	35%	4.8 ± 0.1 *^abcd^*	9.7 ± 0.4 *^abd^*	56.7 ± 0.9 *^cdef^*	23.3 ± 0.8 *^def^*
	30%	5.4 ± 0.3 *^acd^*	10.3 ± 1.1 *^bd^*	64.9 ± 2.4 *^efg^*	27.2 ± 0.2 *^fg^*
**Inulin**	40%	4.1 ± 0.1 *^bc^*	7.0 ± 1.6 *^ac^*	47.4 ± 2.2 *^abc^*	17.9 ± 1.9 *^a^*
	35%	4.9 ± 0.1 *^abcd^*	9.0 ± 0.6 *^abcd^*	59.1 ± 0.9 *^def^*	23.0 ± 0.2 *^cde^*
	30%	5.8 ± 0.4 *^a^*	9.7 ± 1.3 *^abd^*	73.2 ± 7.1 *^g^*	28.3 ± 1.5 *^g^*
	Vacuum Drying 50 °C				
**Maltodextrin**	40%	6.7 ± 0.3 *^ab^*	15.1 ± 0.2 *^d^*	99.8 ± 0.0 *^a^*	42.5 ± 1.6 *^b^*
	35%	4.3 ± 0.1 *^a^*	9.6 ± 0.4 *^abc^*	66.9 ± 1.0 *^bg^*	28.6 ± 0.7 *^ac^*
	30%	4.1 ± 0.2 *^a^*	8.0 ± 1.3 *^a^*	52.4 ± 1.7 *^f^*	24.1 ± 0.9 *^cf^*
**Maltodextrin:Inulin (2:1)**	40%	6.7 ± 0.3 *^ab^*	12.8 ± 0.4 *^bcd^*	96.4 ± 2.8 *^a^*	39.6 ± 1.9 *^be^*
	35%	6.6 ± 0.5 *^ab^*	13.1 ± 1.8 *^bd^*	89.1 ± 3.0 *^ade^*	36.2 ± 2.6 *^de^*
	30%	4.0 ± 0.0 *^a^*	8.3 ± 0.7 *^a^*	54.7 ± 0.6 *^fg^*	23.2 ± 0.4 *^f^*
**Maltodextrin:Inulin (3:1)**	40%	7.5 ± 0.6 *^b^*	13.3 ± 2.3 *^bd^*	97.2 ± 8.5 *^a^*	41.6 ± 1.4 *^a^*
	35%	5.4 ± 0.3 *^ab^*	10.4 ± 1.1 *^abc^*	77.2 ± 0.9 *^bcd^*	32.0 ± 0.4 *^ad^*
	30%	5.6 ± 0.5 *^ab^*	9.6 ± 0.3 *^abc^*	71.8 ± 0.8 *^bc^*	29.5 ± 0.9 *^b^*
**Inulin**	40%	5.2 ± 1.3 *^ab^*	10.5 ± 0.2 *^abc^*	81.5 ± 4.5 *^cde^*	32.3 ± 0.8 *^ad^*
	35%	6.5 ± 1.2 *^ab^*	12.0 ± 0.1 *^abcd^*	94.0 ± 2.9 *^ae^*	38.5 ± 0.7 *^be^*
	30%	5.9 ± 1.4 *^ab^*	8.8 ± 0.1 *^ac^*	71.5 ± 0.9 *^bc^*	28.5 ± 0.7 *^ac^*
	Vacuum Drying 70 °C				
**Maltodextrin**	40%	4.3 ± 0.1 *^bd^*	11.2 ± 1.5 *^abd^*	64.9 ± 4.0 *^ac^*	33.9 ± 2.4 *^bc^*
	35%	4.2 ± 0.3 *^bd^*	9.2 ± 0.1 *^ace^*	57.4 ± 0.1 *^cd^*	28.1 ± 1.4 *^abd^*
	30%	3.1 ± 0.4 *^e^*	7.4 ± 0.5 *^e^*	47.5 ± 3.5 *^d^*	22.5 ± 0.7 *^de^*
**Maltodextrin:Inulin (2:1)**	40%	7.7 ± 0.0 *^g^*	14.6 ± 0.1 *^f^*	104.8 ± 3.9 *^e^*	43.6 ± 0.1 *^f^*
	35%	4.9 ± 0.2 *^ab^*	9.6 ± 0.9 *^abce^*	70.8 ± 2.6 *^ab^*	29.8 ± 0.6 *^ab^*
	30%	5.3 ± 0.3 *^ac^*	10.6 ± 1.1 *^abcd^*	74.6 ± 1.9 *^ab^*	32.2 ± 1.1 *^abc^*
**Maltodextrin:Inulin (3:1)**	40%	5.4 ± 0.2 *^ac^*	11.5 ± 0.0 *^abd^*	72.0 ± 0.8 *^ab^*	31.2 ± 0.3 *^abc^*
	35%	4.7 ± 0.0 *^ab^*	10.6 ± 0.1 *^abcd^*	67.7 ± 0.3 *^abc^*	28.2 ± 1.4 *^abd^*
	30%	3.7 ± 0.1 *^de^*	8.1 ± 0.2 *^ce^*	50.3 ± 0.2 *^d^*	21.3 ± 0.6 *^e^*
**Inulin**	40%	6.1 ± 0.2 *^cf^*	12.2 ± 1.1 *^bdf^*	80.1 ± 1.8 *^b^*	31.8 ± 4.0 *^abc^*
	35%	6.3 ± 0.0 *^f^*	12.7 ± 0.1 *^df^*	79.2 ± 8.1 *^b^*	36.3 ± 0.0 *^c^*
	30%	5.3 ± 0.3 *^ac^*	9.8 ± 0.1 *^abce^*	65.4 ± 0.1 *^ac^*	27.5 ± 0.3 *^ad^*
	Vacuum Drying 90 °C				
**Maltodextrin**	40%	5.0 ± 0.5 *^abc^*	6.0 ± 1.0 *^abc^*	39.3 ± 2.1 *^abc^*	18.2 ± 2.5 *^ad^*
	35%	4.7 ± 0.3 *^ac^*	7.7 ± 0.2 *^ab^*	47.3 ± 0.2 *^cf^*	20.3 ± 0.9 *^de^*
	30%	5.9 ± 0.4 *^ab^*	7.9 ± 0.3 *^b^*	52.6 ± 1.7 *^f^*	23.6 ± 0.7 *^e^*
**Maltodextrin:Inulin (2:1)**	40%	4.1 ± 0.4 *^cd^*	6.1 ± 1.4 *^abc^*	31.3 ± 4.4 *^ade^*	13.4 ± 0.3 *^abc^*
	35%	5.5 ± 0.2 *^ab^*	7.1 ± 0.0 *^abc^*	35.2 ± 1.1 *^abe^*	13.5 ± 1.4 *^abc^*
	30%	6.2 ± 0.8 *^b^*	7.4 ± 1.6 *^ab^*	39.6 ± 2.2 *^abc^*	14.9 ± 1.9 *^abc^*
**Maltodextrin:Inulin (3:1)**	40%	4.6 ± 0.0 *^ac^*	6.7 ± 0.9 *^abc^*	36.3 ± 0.4 *^abe^*	15.5 ± 0.1 *^acd^*
	35%	5.3 ± 0.1 *^abc^*	7.6 ± 0.0 *^ab^*	40.0 ± 0.2 *^abc^*	16.7 ± 0.2 *^ad^*
	30%	5.8 ± 0.0 *^ab^*	7.3 ± 0.6 *^ab^*	43.1 ± 0.6 *^bcf^*	17.0 ± 0.3 *^ad^*
**Inulin**	40%	2.8 ± 0.4 *^d^*	4.4 ± 0.2 *^ac^*	23.5 ± 2.0 *^d^*	9.9 ± 1.0 *^b^*
	35%	3.1 ± 0.2 *^d^*	3.7 ± 0.5 *^c^*	22.0 ± 0.4 *^d^*	11.3 ± 0.9 *^bc^*
	30%	2.8 ± 0.0 *^d^*	4.7 ± 1.4 *^abc^*	28.3 ± 5.6 *^de^*	14.9 ± 2.1 *^abc^*
	Spray Drying				
**Maltodextrin**	40%	3.3 ± 0.1 *^ce^*	7.1 ± 0.7 *^ab^*	42.7 ± 0.9 *^a^*	18.7 ± 1.3 *^a^*
	35%	4.5 ± 0.1 *^ab^*	8.2 ± 0.6 *^acd^*	55.1 ± 0.7 *^bc^*	23.0 ± 2.0 *^abc^*
	30%	5.7 ± 0.5 *^d^*	9.4 ± 0.4 *^cde^*	62.3 ± 1.2 *^cd^*	28.8 ± 0.4 *^d^*
**Maltodextrin:Inulin (2:1)**	40%	2.7 ± 0.2 *^e^*	5.2 ± 0.7 *^b^*	29.3 ± 0.6 *^e^*	12.6 ± 0.6 *^e^*
	35%	3.8 ± 0.6 *^ac^*	6.1 ± 0.1 *^ab^*	44.4 ± 0.0 *^a^*	19.1 ± 1.4 *^ab^*
	30%	5.4 ± 0.1 *^bd^*	7.9 ± 1.4 *^acd^*	60.9 ± 5.0 *^bcd^*	27.1 ± 0.5 *^cd^*
**Maltodextrin:Inulin (3:1)**	40%	3.8 ± 0.3 *^ace^*	6.4 ± 0.9 *^ab^*	45.2 ± 1.7 *^a^*	19.4 ± 0.5 *^ab^*
	35%	4.6 ± 0.1 *^ab^*	8.1 ± 0.2 *^acd^*	53.9 ± 0.3 *^b^*	21.9 ± 0.9 *^ab^*
	30%	5.5 ± 0.2	9.7 ± 0.2 *^de^*	65.6 ± 1.7 *^d^*	27.3 ± 0.7 *^cd^*
**Inulin**	40%	4.1 ± 0.4 *^ac^*	6.9 ± 0.1 *^ab^*	44.9 ± 1.9 *^a^*	18.9 ± 2.1 *^a^*
	35%	4.6 ± 0.0 *^ab^*	7.2 ± 0.2 *^abc^*	56.8 ± 0.1 *^bc^*	23.5 ± 0.0 *^bc^*
	30%	5.4 ± 0.0 *^bd^*	10.9 ± 0.0 *^e^*	66.6 ± 1.7 *^d^*	28.1 ± 1.2 *^d^*

a, b, c, d, e, f, g —different letters within groups stand for different drying methods, i.e., freeze drying, vacuum drying at 50 °C, 70 °C, and 90 °C, and spray drying, and they indicate significant differences (ANOVA, HSD Tukey, *p* ≥ 0.05).

**Table 4:** The content of hydroxymethyl-l-furfural (μg/kg db) in, and the antioxidant capacity (mmol Trolox equivalent/kg db) of, blackcurrant juice powders made with the addition maltodextrin, inulin, and a mixture of maltodextrin–inulin using different drying methods (average ± SD; *n* = 3).

Carrier/Concentration		HMF	TEAC ABTS	FRAP
	Freeze Drying			
**Maltodextrin**	40%	3.6 ± 0.3 *^a^*	31.0 ± 0.3 *^g^*	27.2 ± 0.8 *^e^*
	35%	3.7 ± 0.1 *^ab^*	36.4 ± 1.0 *^a^*	29.9 ± 2.4 *^e^*
	30%	5.4 ± 0.1 *^cd^*	45.8 ± 0.8 *^d^*	39.0 ± 2.5 *^ab^*
**Maltodextrin:Inulin (2:1)**	40%	3.9 ± 0.9 *^ab^*	39.6 ± 0.8 *^b^*	37.9 ± 0.3 *^ab^*
	35%	4.8 ± 0.1 *^abcd^*	42.8 ± 0.5 *^c^*	38.9 ± 1.4 *^ab^*
	30%	5.9 ± 0.1 *^de^*	55.9 ± 1.8 *^f^*	46.1 ± 1.9 *^d^*
**Maltodextrin:Inulin (3:1)**	40%	4.5 ± 0.2 *^abc^*	36.6 ± 0.3 *^a^*	33.8 ± 0.7 *^f^*
	35%	4.8 ± 0.2 *^abcd^*	43.6 ± 2.0 *^cd^*	38.6 ± 1.8 *^ab^*
	30%	5.0 ± 0.2 *^bcd^*	49.8 ± 0.6 *^e^*	43.8 ± 1.0 *^cd^*
**Inulin**	40%	4.4 ± 0.1 *^abc^*	39.8 ± 1.1 *^b^*	35.5 ± 1.2 *^af^*
	35%	5.2 ± 0.2 *^cd^*	49.4 ± 1.1 *^e^*	40.3 ± 1.9 *^bc^*
	30%	6.9 ± 0.4 *^e^*	58.7 ± 1.6 *^f^*	43.8 ± 1.3 *^cd^*
	Vacuum Drying 50 °C			
**Maltodextrin**	40%	9.3 ± 0.4 *^g^*	26.9 ± 2.7 *^e^*	25.1 ± 2.5 *^h^*
	35%	7.4 ± 0.1 *^def^*	35.9 ± 2.4 *^d^*	30.1 ± 2.8 *^f^*
	30%	5.6 ± 0.3 *^abc^*	40.4 ± 2.1 *^a^*	36.8 ± 1.9 *^ab^*
**Maltodextrin:Inulin (2:1)**	40%	6.9 ± 0.4 *^bdef^*	36.6 ± 0.4 *^d^*	33.5 ± 0.8 *^af^*
	35%	6.6 ± 0.5 *^abde^*	47.1 ± 1.6 *^b^*	43.1 ± 0.6 *^dg^*
	30%	5.0 ± 0.1 *^c^*	53.8 ± 1.1 *^c^*	46.9 ± 1.4 *^e^*
**Maltodextrin:Inulin (3:1)**	40%	8.1 ± 0.6 *^fg^*	41.7 ± 1.3 *^a^*	34.8 ± 0.3 *^a^*
	35%	6.3 ± 0.1 *^abd^*	49.2 ± 1.6 *^b^*	40.5 ± 0.9 *^cg^*
	30%	6.1 ± 0.2 *^abc^*	56.9 ± 0.7 *^c^*	44.7 ± 1.5 *^de^*
**Inulin**	40%	5.5 ± 0.2 *^ac^*	40.6 ± 0.6 *^a^*	36.9 ± 0.6 *^abc^*
	35%	7.8 ± 0.5 *^ef^*	46.0 ± 0.2 *^b^*	39.2 ± 0.5 *^bc^*
	30%	6.1 ± 0.0 *^abcd^*	54.7 ± 0.9 *^c^*	45.8 ± 1.4 *^de^*
	Vacuum Drying 70 °C			
**Maltodextrin**	40%	25.0 ± 3.7 *^e^*	22.4 ± 0.9 *^e^*	22.5 ± 0.1 *^d^*
	35%	20.5 ± 0.2 *^d^*	26.2 ± 0.4 *^f^*	25.5 ± 0.3 *^d^*
	30%	13.1 ± 0.7 *^ab^*	39.2 ± 0.1 *^ad^*	33.2 ± 1.6 *^ab^*
**Maltodextrin:Inulin (2:1)**	40%	18.1 ± 0.6 *^cd^*	37.1 ± 1.1 *^a^*	33.6 ± 1.9 *^ab^*
	35%	10.2 ± 0.6 *^a^*	47.0 ± 0.7 *^b^*	40.4 ± 1.3 *^c^*
	30%	9.8 ± 0.4 *^a^*	56.4 ± 2.6 *^c^*	48.6 ± 2.7 *^e^*
**Maltodextrin:Inulin (3:1)**	40%	16.0 ± 0.1 *^bcd^*	37.2 ± 0.6 *^a^*	30.0 ± 0.4 *^b^*
	35%	12.7 ± 0.1 *^ab^*	45.2 ± 0.5 *^b^*	32.1 ± 0.3 *^ab^*
	30%	9.1 ± 0.3 *^a^*	52.8 ± 2.7 *^c^*	42.5 ± 1.9 *^c^*
**Inulin**	40%	17.7 ± 0.4 *^cd^*	41.2 ± 0.8 *^d^*	35.6 ± 0.5 *^a^*
	35%	15.3 ± 0.2 *^bc^*	46.0 ± 2.3 *^b^*	35.3 ± 1.0 *^a^*
	30%	10.2 ± 0.2 *^a^*	53.3 ± 1.7 *^c^*	42.9 ± 2.9 *^c^*
	Vacuum Drying 90 °C			
**Maltodextrin**	40%	22.7 ± 0.7 *^a^*	25.1 ± 0.9 *^f^*	22.9 ± 0.2 *^d^*
	35%	27.2 ± 0.8 *^ab^*	30.0 ± 1.4 *^g^*	25.0 ± 1.8 *^d^*
	30%	36.7 ± 0.3 *^bc^*	38.5 ± 0.5 *^ab^*	32.0 ± 2.0 *^ab^*
**Maltodextrin:Inulin (2:1)**	40%	30.5 ± 2.3 *^ab^*	39.4 ± 0.5 *^ab^*	30.6 ± 1.2 *^ab^*
	35%	45.3 ± 0.4 *^c^*	41.8 ± 0.4 *^b^*	33.2 ± 0.5 *^a^*
	30%	59.3 ± 2.9 *^d^*	48.1 ± 0.6 *^c^*	38.1 ± 2.5 *^c^*
**Maltodextrin:Inulin (3:1)**	40%	29.8 ± 0.8 *^ab^*	34.5 ± 1.2 *^d^*	29.2 ± 1.3 *^b^*
	35%	42.9 ± 0.5 *^c^*	38.1 ± 2.6 *^ae^*	30.6 ± 0.4 *^ab^*
	30%	57.5 ± 1.0 *^d^*	49.5 ± 3.2 *^c^*	40.0 ± 0.8 *^c^*
**Inulin**	40%	59.8 ± 0.7 *^d^*	34.9 ± 0.8 *^de^*	29.7 ± 1.7 *^ab^*
	35%	77.7 ± 5.0 *^e^*	39.7 ± 1.5 *^ab^*	32.8 ± 0.9 *^a^*
	30%	93.4 ± 6.0 *^f^*	49.9 ± 0.5 *^c^*	39.6 ± 2.1 *^c^*
	Spray Drying			
**Maltodextrin**	40%	11.1 ± 0.4 *^e^*	34.4 ± 0.7 *^b^*	32.5 ± 2.2 *^a^*
	35%	13.1 ± 0.6 *^f^*	37.5 ± 0.5 *^ab^*	35.1 ± 1.8 *^ab^*
	30%	17.7 ± 0.3 *^g^*	41.0 ± 2.2 *^a^*	38.4 ± 1.4 *^cd^*
**Maltodextrin:Inulin (2:1)**	40%	4.6 ± 0.0 *^b^*	27.6 ± 0.9 *^e^*	25.9 ± 1.0 *^g^*
	35%	4.7 ± 0.4 *^b^*	40.3 ± 3.1 *^a^*	37.7 ± 3.5 *^abc^*
	30%	9.5 ± 0.4 *^a^*	55.0 ± 0.8 *^d^*	45.2 ± 4.3 *^de^*
**Maltodextrin:Inulin (3:1)**	40%	7.3 ± 0.5 *^c^*	40.7 ± 0.8 *^a^*	37.1 ± 0.9 *^abc^*
	35%	8.8 ± 0.1 *^a^*	48.7 ± 1.0 *^c^*	43.9 ± 1.4 *^de^*
	30%	9.6 ± 0.3 *^ad^*	54.9 ± 2.5 *^d^*	48.8 ± 1.9 *^ef^*
**Inulin**	40%	6.9 ± 0.4 *^c^*	40.8 ± 0.8 *^a^*	36.2 ± 0.7 *^ab^*
	35%	9.5 ± 0.5 *^a^*	47.8 ± 0.9 *^c^*	41.9 ± 1.2 *^cd^*
	30%	11.1 ± 0.1 *^de^*	60.6 ± 1.2 *^f^*	50.7 ± 1.9 *^f^*

HMF—hydroxymethyl-l-furfural; ABTS—Trolox equivalent antioxidant capacity by ABTS; FRAP—ferric reducing antioxidant potential; a, b, c, d, e, f, g —different letters within groups stand for different drying methods, i.e., freeze drying, vacuum drying at 50 °C, 70 °C, and 90 °C, and spray drying, and they indicate significant differences (ANOVA, HSD Tukey, *p* ≥ 0.05).

## Abbreviations

FD	Freeze drying
VD	Vacuum drying
SD	Spray drying
TEAC ABTS	Trolox equivalent antioxidant capacity by ABTS
FRAP	Ferric reducing antioxidant potential
